# The Role of Cardiovascular and Metabolic Comorbidities in the Link between Atrial Fibrillation and Cognitive Impairment: An Appraisal of Current Scientific Evidence

**DOI:** 10.3390/medicina55120767

**Published:** 2019-11-30

**Authors:** Ahmed AlTurki, Jakub B. Maj, Mariam Marafi, Filippo Donato, Giovanni Vescovo, Vincenzo Russo, Riccardo Proietti

**Affiliations:** 1 Division of Cardiology, McGill University Health Center, Montreal, QC H3G1A4, Canada; jakub.maj@mail.mcgill.ca; 2 Department of Neurology and Neurosurgery, Montreal Neurological Institute, Montreal, QC H3A2B4, Canada; mariamarafie@gmail.com; 3 Department of Cardiac, Thoracic, and Vascular Sciences, University of Padua, 35121 Padua, Italy; filippodonato89@gmail.com (F.D.); gm.vescovo@libero.it (G.V.); riccardoproietti6@gmail.com (R.P.); 4 Department of Medical Translational Sciences, University of Campania “Luigi Vanvitelli”-Monaldi Hospital, 80131 Naples, Italy; v.p.russo@libero.it

**Keywords:** atrial fibrillation, metabolic syndrome, obesity, cognitive impairment, dementia

## Abstract

Atrial fibrillation (AF) is the most common arrhythmia encountered in clinical practice with implications on long-term outcomes. Metabolic disorders including diabetes mellitus and obesity are independent predictors of atrial fibrillation and present therapeutic targets to reduce both the incidence and duration burden of atrial fibrillation. The presence of pericardial fat in direct contact with cardiac structures, as well the subsequent release of proinflammatory cytokines, may play an important role in this connection. Atrial fibrillation is an independent predictor of cognitive impairment and dementia. While clinical stroke is a major contributor, other factors such as cerebral hypoperfusion and microbleeds play important roles. New evidence suggests that atrial fibrillation and cognitive impairment may be downstream events of atrial cardiomyopathy, which may be caused by several factors including metabolic syndrome, obesity, and obstructive sleep apnea. The mechanisms linking these comorbidities to cognitive impairment are not yet fully elucidated. A clearer understanding of the association of AF with dementia and cognitive impairment is imperative. Future studies should focus on the predictors of cognitive impairment among those with AF and aim to understand the potential mechanisms underlying these associations. This would inform strategies for the management of AF aiming to prevent continued cognitive impairment.

## 1. Introduction

Atrial fibrillation (AF) is the most common significant arrhythmia and is increasingly prevalent [1]. Hospitalization due to AF has increased by 60% in the United States in the last 20 years and is expected to affect 20 million Americans by 2030 [2]. AF may be complicated by thromboembolic events and heart failure, and it is associated with an increase in mortality [3]. Metabolic disorders including hypertension and diabetes have been shown to significantly increase the risk of these complications in those with AF [4]. In addition, obesity and obstructive sleep apnea have also been associated with AF [5].

Cognitive decline and dementia have emerged as associated risks in patients with atrial fibrillation [6]. A significant portion of this risk is attributable to cerebrovascular thromboembolic events that lead to vascular dementia [7]. However, AF has been shown to be a risk factor for cognitive impairment and dementia independent of stroke [8]. In this review, we will examine the association between metabolic disorders and AF as well as the possible role of these cardiovascular and metabolic comorbidities in the pathogenesis of cognitive impairment in patients with AF.

## 2. Obesity and Metabolic Syndrome

Atrial fibrillation is associated with heart failure, obesity, diabetes, hypertension, and hyperthyroidism [9]. Obesity has been shown to be an independent risk factor for AF [10], and this association has been observed in multiples studies thus far [11,12,13]. Wang et al. [11], analyzed the data of the Framingham Heart Study, a long-standing, multigenerational, longitudinal study of cardiovascular disease, and found that body mass index (BMI) independently predicted AF when adjusted for other risk factors [11]. Each BMI unit increase was associated with a 4% risk increase of AF. Overall, obese men and women had a 52% and 46% greater risk of AF, respectively, when compared to nonobese participants. One of the largest studies to date was a nationwide, prospective cohort from Denmark of 47,589 individuals, where each increase in BMI per unit increased the risk of AF by 8% and 6% in men and women, respectively [14]. In individuals with a BMI over 30, the adjusted hazard ratio for AF was 2.35 in men and 1.99 in women [14]. The association between obesity and AF is independent of ethnicity. In two large cohort studies of 28,449 Japanese individuals and 14,598 American individuals, a significant association between obesity and AF was shown [15,16]. Umetani et al. [17], reported a three-fold risk of AF in individuals with BMI > 25, after adjusting for age and left atrial (LA) size, and that obesity was the strongest metabolic risk factor for AF [17]. In the Atherosclerosis Risk in Communities (ARIC) study, 20% of incident AF could be attributed to obesity. Wanahita conducted a meta-analysis of 16 studies and found a 49% increase in the risk of AF in individuals with a BMI above 30 [13].

Adipose tissue is known to release multiple compounds, many of which are proinflammatory cytokines [18]. Central obesity is strongly associated with insulin resistance [19]. In addition, obesity is associated with the activation of the sympathetic nervous system and renin-angiotensin system, leading to hypertension [20]. These elements define the metabolic syndrome. Metabolic syndrome can practically be considered as a collection of cardiovascular and metabolic imbalances that are associated with a higher risk of developing cardiovascular atherosclerotic disease. Key features include abdominal obesity, dyslipidemia, hypertension, and insulin resistance [21]. Metabolic syndrome has an estimated prevalence of 20% in North America [22], which underlines the need to identify, manage, and prevent potential complications [21].

Metabolic syndrome has been shown to be associated with atrial remodeling and fibrosis, atrial synchronicity, autonomic abnormalities, mitochondrial dysfunction, and increased LA size [23,24]. An electrophysiological study of patients with AF has shown that left atrial low-voltage zones were identified more commonly in patients with metabolic syndrome (46%) than in patients without metabolic syndrome (8.2%). Metabolic syndrome was an independent predictor of left atrial low voltage with 11 times the odds [20]. Each component of metabolic syndrome has been demonstrated to be associated with an increased risk of AF [23]. It is not only the diagnosis of diabetes mellitus that has an association with AF, but increased fasting glucose also independently predicts the risk of AF. Each increase of 18 mg/dL is linked with a 33% increased risk of AF [25].

One mechanism in which obesity may lead to AF is through the presence of pericardial fat, which is of importance because of its contiguity with the heart and a shared blood supply [26]. Pericardial fat is highly metabolically active and releases proinflammatory cytokines. Critically, pericardial fat is more strongly associated with metabolic risk than BMI. Thus, pericardial fat may play a major role in the risk of AF observed with obesity [26].

## 3. Obesity as a Therapeutic Target to Decrease Metabolic Comorbidities

From a pharmacological perspective regarding weight loss, there is little evidence of the benefit in reducing the risk of AF with medications used in diabetes that induce significant weight loss and improve insulin sensitivity, such as glucagon-like peptide -1 receptor agonists, sodium-glucose transport protein 2 (SGLT2), and dipeptidyl peptidase-4 (DPP-4) inhibitors [23]. Osteopontin, a proinflammatory adhesion protein that is upregulated in obese individuals with hypertension, may be a pharmacological target to prevent atrial remodeling [27].

Interventions of weight management have been shown to affect atrial remodeling in a proportional manner and to reduce AF burden. The benefits of weight loss were seen from the structural perspective in a study conducted by Abed and colleagues that showed a significant decrease in the left atrial area and interventricular septal thickness in subjects that lost 14.3 kg, compared to the control group that lost 3.6 kg [28]. In addition, patients who lost significant amounts of weight also experienced a reduction in AF, as well as AF symptom-related frequency and severity [28]. However, there appears to be a minimal threshold of weight loss for AF burden to decrease [23]. The Long-Term Effect of Goal-Directed Weight Management in an Atrial Fibrillation Cohort (LEGACY) study aimed to assess the long-term impact of weight loss on AF rhythm control in patients who were obese, as defined by a BMI of 27 kg/m^2^ [29]. Similarly, in the LEGACY study, subjects with BMI > 27 that lost >10% of body mass had a six-fold decrease of arrhythmias compared to individuals that lost less than 10% of body mass. Patients that lost >5% of body mass, but did not reach the 10% threshold, actually had a two-fold increase in arrhythmia recurrence [29].

Weight control measures that involve medical interventions, rather than exercise or diet, have also been shown to be effective. Bariatric surgery is able to produce significant weight loss that is more sustainable in comparison to nonsurgical interventions [30]. This has been shown to have dramatic effects on metabolic comorbidities, such as hypertension and diabetes mellitus, and may return the patient to a normotensive and normoglycemic state. Reductions in cholesterol have also been noted. This mirrors the effect of weight loss by nonsurgical interventions [28,29]. Interestingly, bariatric surgery has been shown to decrease incident AF as well as reduce the burden of AF in obese individuals [31]. In the Swedish Obese Subjects study, investigators compared 2000 individuals that underwent bariatric surgery to a matched cohort of 2000 patients that received usual care [31]. After a median 19 years follow-up period, incident AF had occurred in 12.4% of patients that underwent bariatric surgery compared to 16.8% of those that received usual care with a 29% relative risk reduction in incident AF [31]. Lynch and colleagues replicated this finding [32].

## 4. Fibrotic Atrial Cardiomyopathy

Metabolic syndrome, which results in hypertension, inflammation, endothelial dysfunction, and myocardial steatosis, leads to left atrial fibrosis and dilatation [23]. Inflammation is the main driver of atrial fibrosis; a proposed mechanism in metabolic syndrome is through the accumulation of intracellular triglycerides and free fatty acids. These deposits appear to be toxic to the atrial myocytes, leading to myocardial apoptosis and fibrosis. The resulting atrial fibrosis leads to structural and electrical remodeling of the left atrium. Atrial dilatation leads to the activation of the renin–angiotensin–aldosterone system, which in itself leads to further myocyte fibrosis, apoptosis, and vasoconstriction by angiotensin II and aldosterone [24]. Atrial fibrosis causes the development of electrical remodeling by delaying interatrial conduction with prolongation of atrial activation time and cycle length, and these changes are enhanced by augmented atrial stretch from obesity. Atrial stretch causes prolongation of the action potential and shortens refractory periods which allow physiological rhythm to be overtaken by reentrant wave fronts from the pulmonary veins, which result in atrial fibrillation. Recurrent episodes of AF enhance atrial remodeling that will promote further events in time.

Atrial cardiomyopathy, therefore, is hypothesized to be the result of these multiple metabolic derangements including hypertension, diabetes, and metabolic syndrome [33]. In turn, AF may also independently be associated with strokes and cognitive decline. This notion is supported by findings seen in patients with subclinical AF in whom studies found a significant increase in stroke risk [34]. The classical theory is that AF causes blood to stagnate in the left atrium that leads to a stroke. However, data from patients with subclinical device-detected AF put this theory into question. There was no temporal relationship between AF episodes and stroke events, with the majority of AF episodes occurring >30 days prior to the stroke event [35,36]. This lends credence to the notion that atrial hypocontractility and impaired atrial endothelial function in the context of atrial cardiomyopathy contribute significantly to stroke events and do not require the presence of AF [33]. In a recent consensus document by the European Heart Rhythm Association that examined the current literature on the subject, hypertension, obesity, and diabetes mellitus were noted to lead to atrial cardiomyopathy through the promotion of changes to the cardiac myocyte, fibrosis, and noncollagenous infiltration [37].

## 5. Atrial Fibrillation (AF) and Cognitive Function

Both atrial fibrillation and dementia are common diseases that share similar risk factors, but the association between them appears to be independent of shared risk factors. Table 1 summarizes the current body of literature demonstrating the association between AF and dementia. An improved understanding of the predictors of cognitive impairment in patients with AF, and the potential underlying mechanisms, is critical for the management of AF with the aim to prevent adverse outcomes in these patients [38].

Several studies have documented the association of AF and cognitive impairment. In 935 participants of the ARIC study with no prior history of strokes, incident AF was associated with a more rapid decline in executive function and verbal fluency [39]. The association was only found in those with subclinical strokes, which suggested a thromboembolic cause of the association. Ott and colleagues assessed data from a cross-sectional study with 6584 participants, of whom 9.6% had cognitive impairment and 4.2% had dementia [40]. The most common causes of dementia were Alzheimer’s disease (75%), vascular dementia (15%), and undefined dementia (11%). Participants with AF had twice the odds of having dementia compared to those without AF.

Other studies have shown that the association between AF and cognitive decline goes beyond thromboembolic disease. Marzona and colleagues performed a combined post hoc analysis of two prospective multicenter trials, Telmisartan, Ramipril, or Both in Patients at High Risk for Vascular Events (ONTARGET) and The Telmisartan Randomised Assessment Study in ACE intolerant subjects with cardiovascular Disease (TRANSCEND), which comprised 31,506 patients aged 55 years and older with cardiovascular disease or diabetes [41]. At baseline, 3.3% had AF, while 6.5% developed AF during follow-up (median 56 months). AF was associated with a 14% increased risk of cognitive decline and a 16% increased risk of new-onset dementia; these results were independent of any history of overt stroke [41]. Santangeli published a systematic review of eight prospective studies that included over 77,000 patients. The risk of dementia in AF patients was increased by 40% (hazard ratio (HR) 1.42, 95% confidence interval (CI) 1.17–1.72, *p* = 0.002) [6]. Kim and colleagues analyzed data from a large population-based cohort. The association between incident AF and subsequent incident dementia was assessed in 262,611 participants aged 60 years and older that were free of stroke and dementia in Korea between 2005 and 2012. Incident AF was observed in 10,435 participants over a time frame of 1,629,903 person-years at a rate of 0.64% per year. During that time period, incident dementia occurred at a rate of 4.1 and 2.7 per 100 person-years in those with incident AF and a propensity score-matched AF-free group, respectively. Incident AF increased the risk of dementia by 50% (HR 1.52; 95% CI 1.43–1.63). Incident AF was associated with an increased risk of both Alzheimer’s (HR 1.31, 95% CI 1.20–1.43) and vascular dementia (HR 2.11, 95% CI 1.85–2.41). Oral anticoagulation in patients who developed incident AF was associated with a reduced risk of incident dementia (HR 0.61, 95% CI 0.54–0.68), while higher CHA2DS2-VASc scores were associated with an increased risk of dementia [42].

AF is associated with an increased risk of progressive cognitive impairment in patients who have not suffered from a stroke. In a secondary analysis of the Cardiovascular Health Study, patients with AF had a more rapid decline of cognitive function compared to patients with sinus rhythm [43], as assessed by the Mini Mental State Exam. Cognitive impairment risk is greater in AF patients with heart failure, diabetes, and kidney disease [44]. A higher risk of dementia in patients with AF that have not suffered from strokes is found in both men and women. In a large study of 35,608 patients without a history of AF or dementia, of whom 40.4% were women, the five-year rates of AF were higher in men than women (14.0% in men versus 11.9% in women; *p* < 0.0001). However, dementia rates (1.1% in women versus 0.9% in men; *p* = 0.09) were similar in women and men. Among the patients who developed AF, the five-year rate of dementia in women was 2.9%, versus 2.3% in men (*p* = 0.180) [45].

Multiple mechanisms of cognitive dysfunction due to atrial fibrillation have been postulated. The most intuitive mechanism is ischemic stroke causing cognitive decline. The risk of ischemic stroke is four to five times greater in individuals with atrial fibrillation. Subclinical strokes contribute significantly to cognitive decline. AF is significantly associated with subclinical strokes, which has been shown to confer a two- to seven-fold increase in odds [8]. Chronic brain hypoperfusion is believed to be a second mechanism of cognitive decline in AF. Cardiac output can decrease as a result of beat-to-beat variability, leading to hypoperfusion and hypoxia of the brain. Animal models have shown that hypoperfusion of the brain reduces clearance of amyloid beta, which may lead to Alzheimer’s dementia [7]. In a modeling analysis performed to assess the hemodynamic effect of AF on brain perfusion, the variance in R-R intervals as well as loss of atrioventricular synchrony led to a reduction in cerebral blood flow that led to repetitive hypoperfusions [46]. Lastly, systemic inflammation is increased in patients with AF, as evidenced by elevated markers such as C-reactive protein and tumor necrosis factor and may lead to cognitive impairment via cerebrovascular dysfunction [7]. Systemic inflammation may cause cerebral microinfarction with subsequent cognitive dysfunction via endothelial dysfunction, tissue factor release, and platelet activation [38]. Intensive lipid-lowering treatment with 40 mg atorvastatin and 10 mg ezetimibe, which has been shown to have anti-inflammatory properties, slows neurocognitive deterioration and cortical volume loss [47].

**Table 1:** Important studies in the association between atrial fibrillation and dementia.

Study First Author (Year)	Study Details	Outcomes
Bunch et al., [48]	Prospective database 3-year follow-up 16,848 with AF and 16,848 age/gender matched controls without AF.	0.9% of the AF patients and 0.5% of the no AF patients
Dublin et al., [49]	Prospective cohort study. A population-based sample of 3045 community-dwelling adults aged 65 and older without dementia or clinical stroke followed from 1994 to 2008. AF identified using codes	572 participants (18.8%) developed dementia (449 with Alzheimer’s disease). The adjusted hazard ratio associated with AF was 1.38 (95% confidence interval (CI) = 1.10–1.73) for all-cause dementia and 1.50 (95% CI = 1.16–1.94) for possible or probable Alzheimer’s disease).
De Bruijn et al. [50]	Prospective cohort study 6514 dementia-free participants in the prospective population-based Rotterdam Study 20 years of follow-up Clinical criteria	Incident AF was associated with an increased risk of dementia in younger participants (<67 years: 1.81; 1.11–2.94 vs. ≥67 years: 1.12; 0.85–1.46; *p* = 0.02 for interaction)
Ding et al. [51]	Prospective cohort study 2685 dementia-free participants from the Swedish National Study on Aging and Care who were regularly examined from 2001–2004 to 2010–2013. 9 years of follow-up Clinical criteria	AF was significantly associated with an increased risk of all-cause dementia (HR = 1.40, 95% CI: 1.11–1.77) and vascular and mixed dementia (HR = 1.88, 95% CI: 1.09–3.23)
Marzona et al. [41]	Post-hoc analysis of two randomized controlled trials, TRANSCEND and ONTARGET 31,506 participants 56 months follow up Clinical outcomes	AF was associated with an increased risk of incident dementia (HR 1.30, 95% CI 1.14–1.49)
Rusanen et al. [52]	2000 participants who were randomly selected from four separate, population-based samples originally studied in midlife 25 year follow up Clinical outcomes	AF in late-life was an independent risk factor for dementia (HR 2.61, 95% CI 1.05–6.47; *p* = 0.039) and AD (HR 2.54, 95% CI 1.04–6.16; *p* = 0.040)

## 6. Future Directions

Given the increased prevalence of AF, metabolic syndrome, and cognitive impairment, it is imperative that our understanding of the associations and interactions of these entities also increases. Prospective studies with well-defined and adjudicated predictors and outcomes are needed. This includes clear criteria for the subdivisions of dementia and prolonged screening for AF prior to the commencement of studies. New technology that allows continuous ambulatory monitoring of AF will significantly increase our ability to screen for AF. The Apple Heart Study, which enrolled 419,093 participants, disclosed the preliminary findings that 2161 participants (0.5%) received a pulse notification for AF. These patients were then invited to wear an electrocardiogram patch, and AF was identified in 34% of participants [53] Studies assessing the efficacy of a variety of treatment modalities for prevention and treatment of cognitive impairment in the context of AF will inform future clinical practice. Ongoing trials such as Blinded Randomized Trial of Anticoagulation to Prevent Ischemic Stroke and Neurocognitive Impairment in AF (BRAIN-AF) (NCT02387229) will assess whether anticoagulation is effective in patients without traditional risk factors for stroke. Ongoing improvement in imaging may allow further characterization of cerebral changes and explain mechanisms of the association between AF and dementia.

## 7. Conclusions

AF is associated with cognitive impairment and dementia via stroke-dependent and independent mechanisms. Metabolic comorbidities are independent predictors of AF and may be a cause of atrial cardiomyopathy, which would in turn contribute to AF as well as non-stroke-related mechanisms. Further studies are required to elucidate and identify predictors of this association. Such knowledge may inform future management of AF.
